# Combine Your “Will” and “Able”: Career Adaptability’s Influence on Performance

**DOI:** 10.3389/fpsyg.2018.02695

**Published:** 2019-01-22

**Authors:** Xueyuan Gao, Xun Xin, Wenxia Zhou, Denise M. Jepsen

**Affiliations:** ^1^Faculty of Business and Economics, Macquarie University, Sydney, NSW, Australia; ^2^School of Labor and Human Resources, Renmin University of China, Beijing, China; ^3^Business School, Southwest University of Political Science & Law, Chongqing, China

**Keywords:** career adaptability, proactive personality, career self-management, performance, career construction theory

## Abstract

Adaptivity and adaptability are two key elements representing one’s “willingness” and “ability,” respectively, in the career construction theory (CCT) framework. On the basis of CCT and complemented by the visual of resources in the conservation of resources theory, this study combines career issues and performance and examines the joint effect of adaptivity and adaptability on career self-management which will lead to improved performance. Using a sample of Chinese employees (*N* = 232), the study first examines the mediating role that career self-management plays between career adaptability and performance and then tests the moderating role of proactive personality. Results show career adaptability positively predicts performance, with this relationship partially mediated by career self-management. The positive effect of career adaptability on career self-management is stronger among those who are more proactive than less proactive. Further, the indirect effect of career adaptability on performance is stronger among proactive employees than those with lower levels of proactive personality. These findings provide implications for both theories and practices.

## Introduction

Performance is a concept that has been valued by companies for a long time, yet good performance largely depends on the endeavor of individuals. Employees in a company are creators of organizational performance and are also the principle of their own career development. Individuals are increasingly more flexible in making career choices and more likely to have more career self-management behaviors than in the past to influence their career development (Lent et al., 2016). The dual identity of individuals in organizations highlights the importance of combining career issues and performance fields. Only when organizations give more attention to individuals’ career development, can they put more effort into their work which thus leads to good work results. In the present study, we examine performance through the lens of individual career issues.

Career construction theory (CCT) (Savickas, 1997, 2005, 2013), also called the adaption model of career construction, provides a way to connect career issues and performance and helps to explain how career issues will promote individual performance. Career adaptability as a central concept in CCT refers to an individual’s psychological resources and represents one’s ability toward work tasks. The other three components in the CCT framework are adaptivity, adapting responses and adaptation results. Specifically, adaptivity is a trait-like and stable psychological characteristic involving one’s readiness and willingness, to adapt to a change in career. Individuals’ adaptivity can be measured through their cognitive ability, proactive personality or the big five personality traits (Savickas and Porfeli, 2012). “Adaptivity positively influences career adaptability, which in turn positively influences adapting responses and adaptation results (Rudolph et al., 2017).” Adapting responses are the beliefs or behaviors of individuals on how to deal with career development tasks (Hirschi et al., 2015). Operational indicators of adapting responses include behaviors such as career self-management or career planning (Rudolph et al., 2017). Adaptation results mostly refers to the suitability between a person and their surroundings. Goals of career adaptability are to achieve adaptation results that are indicated by individual development, satisfaction or career success with performance included (Savickas and Porfeli, 2012; Savickas, 2013).

As for the effect that proactive personality has on career adaptability in the CCT framework, numerous studies to date have largely examined proactive personality’s influence on career adaptability. For example, in early empirical studies, Savickas (2005) and Duffy (2010) found the significant positive effect of proactive personality on career adaptability. Tolentino et al. (2014) then showed that the students’ career adaptability can be positively influenced by proactive personality. Additionally, in resent work, proactive personality is continuously being examined as a positive antecedent of ability (Uy et al., 2015; Nilforooshan and Salimi, 2016; Guan et al., 2017). Despite the largely tested cause and effect relationship between proactive personality and career adaptability, the CCT also addressed the interplay between the two. In particular, “higher levels of adaptation (outcome) are expected for those who are willing (adaptive) and able (adaptability) to perform behaviors” (Savickas and Porfeli, 2012, p. 663). Thus, high levels of career self-management require both proactive personality and career adaptability which will then lead to good performance. Concurrently, based on the conservation of resources theory (COR, Hobfoll, 2001), both ability and personality are identified as different categories of individual resources. Career adaptability is a type of volatile resource, which can be more easily changed or transferred than proactive personality which works as a stable key personal resource (Brummelhuis and Bakker, 2012). The combination of different resources promote each other and lead to good results. Therefore, proactive personality works, on the one hand, as a predictor of career adaptability, which has been tested in prior studies. On the other hand, as a distinctive stable trait factor, proactive personality can also interplay with career adaptability which leads to adaptation results. Yet up to now, there are few studies examining the joint effect of career adaptability and proactive personality in the CCT framework. The exploration of proactive personality as a boundary condition is meaningful, providing a new lens under which to test the CCT framework.

**FIGURE 1:**
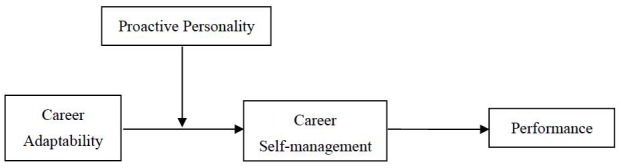
The proposed moderated mediation model.

To provide a summary, the goals of this study are (1) to explain how individuals’ career adaptability will promote their performance from a CCT perspective and (2) to investigate the mechanism and boundary conditions that lies in the relationship between career adaptability and performance. At the same time, our study aims to present and test an extended relationship in the CCT. On the one hand, points in the CCT framework have addressed the importance of combining proactive personality and career adaptability (Savickas and Porfeli, 2012), yet few studies, in practice, have taken this into consideration. On the other hand, the resource perspective of the COR theory (Hobfoll, 1989) can further complement the CCT and provide supportive evidence of the joint effect between proactive personality and career adaptability. Therefore, in the following sections, we hypothesize and investigate the interaction effects of career adaptability and proactive personality, on career self-management and performance among Chinese employees. As shown in Figure 1, we first examine the direct effect from career adaptability to performance. The mediation effect of career self-management is then tested. We inspect proactive personality as a moderator affecting the relationship between career adaptability and career self-management, before testing the final moderated mediation model.

This research has two main contributions. First, the mediation effect of career self-management, when career adaptability predicts performance, is examined in the CCT (Savickas, 1997, 2005, 2013) framework. We therefore related performance, which is most valued by organizations, with career issues which are most valued by individuals, and tightly combined the benefits of organizations and employees. Second, we theorized and examined the moderating role that proactive personality plays, by inserting the visual of COR (Hobfoll, 1989) on resources, into the CCT framework. In doing so we found a link between the CCT and COR, as the two theories both regard proactive personality as an important component of personal resources. We also addressed the need for comprehensive knowledge of the CCT model. Specifically, other than a simple linear cause and effect relationship, proactive personality can also interact with career self-management in predicting career self-management. Further, high levels of career self-management will lead to a good performance. We will therefore follow with the development our hypotheses first and then discuss the core findings, as well as the implications from both a theoretical and practical perspective.

### Literature Review and Hypotheses Development

#### Career Adaptability, Career Self-Management and Performance in the Career Construction Theory Framework

Drawing from the CCT (Savickas, 1997, 2005, 2013), we inform our study on how the three variables – career adaptability, career self-management and performance – can be integrated as operational indicators of adaptability resources, adapting responses as well as adaptation results separately for the study. Career adaptability refers to “a psychosocial construct that denotes an individual’s resources for coping with current and anticipated tasks, transitions, or traumas in their occupational roles” (Savickas and Porfeli, 2012). Career adaptability is known as a psycho-social resource that represents self-regulatory capacities and can lead to various adapting behavioral responses and adaptation results. Career adaptability has been examined as a higher-order construct in previous studies. It contains four dimensions, including “concern, control, curiosity and confidence” (Savickas and Porfeli, 2012, p. 662). Career concern represents individuals’ ability to foresee and predict the occurrence of an event that might lead to a change in work tasks in the future. Career control represents individuals’ self-discipline, which may affect their ability to take conscientious action. Career curiosity entails individuals’ ability to identify career opportunities and explore the relationship between themselves and their surroundings. Career confidence is a positive belief to overcome difficulties when pursuing career goals. Individuals with strong career adaptability will be willing and able to invest their inner resources, such as physical or emotional energies, into their career development within these four dimensions (Savickas, 2013; Guo et al., 2014). In contrast, those who do not possess career adaptability will doubt themselves and show little confidence toward their career.

Career self-management is an operational indicator of adapting responses within the adaption model of career construction (Savickas, 1997). According to the CCT, career self-management can be encouraged by career adaptability (Rudolph et al., 2017). Research suggests that career self-management involves the three career self-managing behaviors of “positioning, influence and boundary management” (King, 2004, p. 127). Positioning behaviors pertain to the skills or experience to realize career goals. Influence behavior refers to the influencing effect that individual activities have on the organizational decision-making process. Boundary management concerns the balance of work demands and non-work domains. Employees regularly use career self-managing behaviors for gathering information, planning to solve problems and making decisions during the career process, to achieve desired career outcomes (Kossek et al., 1998). As a way of overcoming difficult situations that would frustrate career progression, career self-management is important in an individual’s career development (Crites, 1976).

Work performance is a key indicator of objective career success (Rosikiewicz et al., 2016) and an operational indicator of adaptation results in the CCT (Savickas, 2013; Rudolph et al., 2017). Performance in this study refers to general task performance (Borman and Motowidlo, 1993) in work situations and how one can do work to his/her best ability along with dealing with workplace relationships. Many factors can influence performance, such as personal resources or employee-organization relationships (Tsui et al., 1997). For example, Ohme and Zacher (2015) demonstrated that an individual’s career adaptability can lead to good performance, and Paradnike and Akkermans (2017) found that career adaptability can enhance study success via study engagement.

#### The Mediating Role of Career Self-Management

Career self-management represents a individuals’ initiative, activities, and behaviors (King, 2004). Individuals who are good at managing their career are more likely to have high degrees of personal initiative and be better at dealing with career development tasks, compared to those with lower levels of career self-management (Frese and Fay, 2001; Hirschi et al., 2015). Career self-management is a career related behavior that can bring individuals and their organizations together to the benefit of both sides. Good career self-management can result in frequent salary progression and promotions (Tharenou, 1997; Brummelhuis and Bakker, 2012), good task performance for individuals and can positively influence organizational effectiveness to differing degrees (Motowidlo and Van Scotter, 1994; Rotundo and Sackett, 2002). Moreover, in line with the adaption model of career construction, career self-management, as an adapting behavior, can be encouraged by career adaptability. The positive effect of career adaptability on employability, as well as work engagement, has been tested before (Rossier et al., 2012). When an individual has a strong employability and is highly engaged in work, due to high levels of career adaptability, work performance will be expectedly increased. Additionally, work performance is also an indicator of the fit between individual and their working environments and the goodness of fit is addressed as an adaptation result in the CCT framework. Therefore, in accordance with the CCT framework (Savickas and Porfeli, 2012; Savickas, 2013), career self-management could be encouraged by career adaptability and can mediate the association between career adaptability and performance.

In sum, based on the CCT (Savickas, 1997, 2005, 2013), individuals will rely on their career adaptability resources to generate a specific career self-management behavior to achieve good performance and to attain a person-environment fit. Thus, we propose a mediation model between career adaptability, career self-management and performance:

Hypothesis 1a. Career adaptability can positively predict performance.
Hypothesis 1b. Career self-management plays a mediating effect between career adaptability and performance.

#### Role of Proactive Personality

The interplay effect of proactive personality and career adaptability, in predicting career outcomes, is of great interest in our research model. Proactive personality is a type of individual disposition, aiming at identifying opportunities and acting to influence the surroundings. Research shows that proactive personality is “one who is relatively unconstrained by situational forces and who effects environmental change” (Bateman and Crant, 1993, p. 105). People who are proactive will show a willingness and confidence to take risks as well as a desire to achieve (Bateman and Crant, 1993; Crant, 2000). They are also likely to be self-initiated and will focus on developing themselves (Parker et al., 2010). A meta-analysis found that proactive personality can predict objective career success, such as job performance, which was stronger than any other personality trait, including the Big Five factors (Thompson, 2005; Fuller and Marler, 2009). Other studies have found that proactive personality can positively affect subjective career success, such as career satisfaction (Jawahar and Liu, 2016; Turban et al., 2017).

The CCT recognizes that adaptivity can lead to adaptability. Thus, proactive personality, an operationalized indicator of adaptivity, can positively predict career adaptability. Research shows that proactive individuals can prepare well to manage career tasks and changes (Rudolph et al., 2017). There are many works examining proactive personality as a predictor of career adaptability (Duffy, 2010; Buyukgoze-Kavas, 2016). Extending the CCT (Savickas, 1997, 2005, 2013) view on the relationship between proactive personality and career adaptability, from the visual of COR (Hobfoll, 1989) on resources, this study examines how proactive personality interacts with career adaptability.

Given that people who have high levels of proactive personality tend to perform proactive work behaviors, we suspect that under the same level of career adaptability, those who are more proactive, intend to be more active in exploring and manipulating their surroundings than less proactive personalities would. Moreover, COR (Hobfoll, 1989) suggests proactive personality as a trait-like key resource, which is stable in a person, while career adaptability is a volatile resource with more flexible characteristics. Different kinds of resources can be combined in different ways and then impact individual behavior differently. For example, some people may have strong adaptability but not active enough because of the lack of autonomy or other environmental support, while others, with the same level of career adaptability, are also very proactive toward work. The compiled effectiveness of different resources will be greater than the effect of a single one.

As a consequence, it is reasonable to suspect that proactive personality will have an amplification effect with respect to the link of career self-management with career adaptability.

Hypothesis 2. Proactive personality will moderate the relationship between career adaptability and career self-management, such that under higher as opposed to lower levels of proactive personality, career adaptability will have a stronger effect on career self-management.

Considering the mediating effect that career self-management plays between career adaptability and performance, we argue that proactive personality will moderate the indirect effect that career adaptability plays on performance. Thus, we propose that:

Hypothesis 3. Proactive personality will moderate the indirect effect of career adaptability on performance, such that compared with the low proactive group, positive effects of career adaptability on performance, through career self-management, will be greater for those with higher levels of proactive personality.

## Materials and Methods

### Procedure and Participants

Data was gathered from a Chinese manufacturing company with branches in Beijing, Tianjin, Shanghai and Shenzhen in 2016. The data collecting procedure included three steps. Specifically, we first obtained permission from the management group to conduct an online survey of their nearly 2000 employees. An invitational email was sent to all employees in the four branches of the company, and we received a total of 301 volunteers. An email with written informed consent along with the survey link was then sent to all volunteers, with a shopping coupon as an incentive. They were asked to submit the survey within a week. All surveys were anonymous, and volunteers could fill the surveys in during or after working hours. There were 232 valid responses representing a response rate of 77% among all original 301 volunteers. Respondents were 139 (60%) males and 93 (40%) females, with an average age of 32.7 years (*SD* = 5.27). Most (84%) had been employed in their current job for 1–4 years. Nine (3.9%) respondents’ education level was below high school level, 25 (10.8%) hold associate degrees, 168 (72.4%) hold bachelor’s degrees, 30 (12.9%) hold master’s degrees or above. The sample included a variety of occupations, including 52 (22.4%) administration, 24 (10.3%) production, 29 (12.5%) research, 48 (20.7%) sales, 32 (13.8%) and finance positions, and 47 (20.3%) other occupations (e.g., logistics support).

### Measures

#### Career Adaptability

Career adaptability was measured by the Career Adapt-Abilities Scale (Hou et al., 2012; Savickas and Porfeli, 2012) with 24-items. The scale has four dimensions, of the four aspects of career adaptability, and each dimension contains six items. Within the stem of career adaptability, sample items for each dimension were “Thinking about what my future will be like,” “Making decisions all by myself,” “Looking for opportunities to grow,” and “Overcoming difficulties.” Respondents rated statements from “Strongly disagree” to “Strongly agree,” indicated from 1 to 5, on a five-point Likert scale. Cronbach’s alpha for the total career adaptability scale was 0.94. Cronbach’s alpha for each sub-scale were 0.85, 0.83, 0.86 and 0.85.

#### Career Self-Management

Career self-management was measured using an 11-item scale developed for Chinese participants (Weng and McElroy, 2010) based on previous research (Noe, 1996; Zikic and Klehe, 2006). The scale consists of three sub-dimensions: career exploration, development of career goals and career strategy implementation. A sample item is “I have developed a detailed career development plan.” Respondents rated statements from “Strongly disagree” to “Strongly agree,” indicated from 1 to 5, on a five-point Likert scale according to their actual career self-management experiences. The scale’s Cronbach’s alpha was 0.86.

#### Proactive Personality

We measured proactive personality with the 10-item proactive personality scale developed by Bateman and Crant (1993). The scale was translated from English into Chinese following a strict translation procedure by two doctoral candidates majoring in English. Respondents rated statements from “Strongly disagree” to “Strongly agree,” indicated from 1 to 5, on a five-point Likert scale according to the extents of their agreement. Sample items included “Nothing is more exciting than seeing my ideas turn into reality” and “Wherever I have been, I have been a powerful force for constructive change.” The scale’s Cronbach’s alpha was 0.87.

#### Performance

Performance was measured by the Chinese version of a four-item scale that was developed and translated from English under the translation and back-translation procedure by Chen et al. (2002). A sample item was “I can finish my work on time.” Respondents rated statements from “Strongly disagree” to “Strongly agree,” indicated from 1 to 5, on a five-point Likert scale according their situations. The Cronbach’s alpha for the performance scale was 0.91.

#### Control Variables

To make our model testing more accurate, we used some factors as control variables. Specifically, we controlled for gender (0 = male, 1 = female) because evidence suggests that males have higher capability beliefs than females (Hirschi, 2009). We controlled for education and length of service (dummy coded, 1 year and below as reference group) as these variables have previously been found to influence career outcomes (Zacher, 2014; Rudolph et al., 2017).

## Results

### Confirmatory Factor Analysis and Common Method Variance

To evaluate the distinctiveness of all variables in the current study, the confirmatory factor analysis (CFA) was first conducted before the analysis with the Mplus7.0. In particular, we evaluated our research model against other competing models. The CFA results indicated that a four-factor model distinguishing between career adaptability, career self-management, performance and proactive personality was a better fit to the data (χ^2^ = 2254.84; *df* = 1028; *p* < 0.001; RMSEA = 0.07; TLI = 0.88; CFI = 0.89; SRMR = 0.07) than other plausible models: (a) a three-factor model combining career adaptability and career self-management in one factor (χ^2^= 2673.18; *df* = 1031, *p* < 0.001; RMSEA = 0.08; TLI = 0.72; CFI = 0.74; SRMR = 0.08); (b) a three-factor model combining career adaptability and performance in one factor (χ^2^ = 2461.24, *df* = 1031, *p* < 0.001; RMSEA = 0.07; TLI = 0.72; CFI = 0.73; SRMR = 0.08); (c) a three-factor model combining career adaptability and proactive personality in one factor (χ^2^ = 2427.09, *df* = 1031, *p* < 0.001; RMSEA = 0.08; TLI = 0.84; CFI = 0.86; SRMR = 0.08) and (d) a one-factor model in which all variables in our study loaded on one factor (χ^2^ = 2962.78, *df* = 1034, *p* < 0.001; RMSEA = 0.09; TLI = 0.62; CFI = 0.64; SRMR = 0.09). Results of the CFA showed that all concepts in our study are clearly distinctive and the respondents that we surveyed could differentiate different latent variables.

To further test the common method variance in our study, we used the method of controlling for effects of an unmeasured latent methods factor (Podsakoff et al., 2003) which was conducted using the Mplus 7.0. Results showed that after adding a common method factor to the four-factor model, the new five-factor model [χ^2^(982) = 1956.219, TLI = 0.90, CFI = 0.91, RMSEA = 0.07, SRMR = 0.09] did not have a better fit than the four-factor model [Δχ^2^ = 298.62, Δ*df* = 46, *p* < 0.05; ΔTLI = 0.01, ΔCFI = 0.02; ΔRMSEA = 0.000, ΔSRMR = 0.02]. In particular, the SRMR index in the five-factor model even outnumbered the upper limit standard (i.e., SRMR = 0.08). After the common method factor was added, the model did not have a better fit, therefore, the analysis results informed our study that the common method bias in our data was acceptable.

### Descriptive Statistics

The descriptive results are shown in Table 1, with the reliability coefficients shown in the brackets. Results showed that career adaptability positively correlated with proactive personality (*r* = 0.73, *p* < 0.01), career self-management (*r* = 0.41, *p* < 0.01) and performance (*r* = 0.32, *p* < 0.01). Proactive personality positively correlated with career self-management (*r* = 0.51, *p* < 0.01) and performance (*r* = 0.41, *p* < 0.01). Career self-management correlated positively with performance (*r* = 0.50, *p* < 0.01). These results (in the expected direction) support the positive effects that career adaptability and career self-management have on performance.

**Table 1:** Descriptive statistics, reliability coefficients, and inter-correlations among variables.

	Mean	*SD*	1	2	3	4	5	6	7	8	9	10	11
1 Gender	1.40	0.49	–										
2 Education	2.94	0.62	-0.10	–									
3 Length of service	1.93	0.90	-0.01	-0.05	–								
4 Career concern	4.09	0.69	-0.04	0.11	-0.02	(0.85)							
5 Career control	4.05	0.66	-0.06	0.04	-0.06	0.70^∗∗^	(0.83)						
6 Career curiosity	3.87	0.70	-0.06	-0.02	-0.06	0.72^∗∗^	0.69^∗∗^	(0.86)					
7 Career confidence	4.00	0.65	0.01	-0.06	0.04	0.66^∗∗^	0.66^∗∗^	0.72^∗∗^	(0.85)				
8 Career adaptability	4.00	0.59	-0.04	0.02	-0.03	0.88^∗∗^	0.87^∗∗^	0.90^∗∗^	0.86^∗∗^	(0.94)			
9 Proactive personality	3.73	0.59	-0.05	0.04	-0.04	0.60^∗∗^	0.61^∗∗^	0.66^∗∗^	0.69^∗∗^	0.73^∗∗^	(0.86)		
10 Career self-management	2.83	0.52	0.08	0.06	-0.13	0.38^∗∗^	0.32^∗∗^	0.41^∗∗^	0.31^∗∗^	0.41^∗∗^	0.51^∗∗^	(0.87)	
11 Performance	3.48	0.53	0.05	-0.06	0.04	0.20^∗∗^	0.28^∗∗^	0.31^∗∗^	0.34^∗∗^	0.32^∗∗^	0.41^∗∗^	0.50^∗∗^	(0.91)

*Reliability coefficients appear in brackets on the diagonal. ^∗∗^p < 0.01.*

### Hypotheses Testing

Following the moderation mediation model examining procedure (Preacher and Hayes, 2008), we examined how career adaptability impacted on performance and whether career self-management played a mediating role between career adaptability and performance. There are three criteria that should be met to examine the mediation role. First, the independent variable should have a significant relationship with the mediator. Second, besides the effect that independent variables play on the outcome, the mediator should significantly predict the outcome. Finally, the indirect effect should be significant. Prior to the analyses, according to the suggestions of Aiken et al. (1991), we centered all continuous variables to better explain the regression model in our study (Aiken et al., 1991).

Results from Table 2 show that when controlling for the effects of gender, education and length of service, career adaptability (*B* = 0.29, *p* < 0.001) was positively related to performance (as shown in Model 4 in Table 2). Hypothesis 1a is therefore supported. The mediation effect that career self-management plays between career adaptability and performance was then tested. After entering career self-management into the regression in Model 5, the results indicate a positive relationship between career self-management and performance (*B* = 0.47, *p* < 0.001). Career adaptability’s coefficient decreased from 0.29 (*p* < 0.001) to 0.12 (*p* < 0.05). We further used the PROCESS program in SPSS software (Hayes, 2013, 2017) to analyze the indirect effect that career adaptability plays on performance, through career self-management. Results show that the indirect effect is also significant (95% CI = [0.09, 0.27]). Therefore, the effect that career adaptability plays on performance is partially mediated by career self-management. Hypothesis 1b is therefore supported.

**Table 2:** Hierarchical regressions: career self-management and performance as outcomes.

Predictors	Career self-management			Performance			
	Model 1	Model 2	Model 3	Model 4	Model 5	Model 6	Model 7
Constant	1.227	2.679	2.56	2.343	1.769	3.436	2.439
Gender	0.109	0.12^*^	0.15^**^	0.06	0.02	0.09	0.02
Education	0.04	0.04	0.04	-0.05	-0.07	-0.05	-0.07
Length of service	-0.06^*^	-0.06^*^	-0.07^*^	0.03	0.05	0.02	0.05
Career adaptability	0.36^***^	0.10^*^	0.19^*^	0.29^***^	0.12^*^	0.12^+^	0.05
Proactive personality		40^∗∗∗^	0.38^***^			0.33^***^	0.18^*^
CA × PP			0.24^***^			0.10	0.06
Career self-management					0.47^***^		0.39^***^
*Adjusted R^2^*	0.18	0.26	0.34	0.10	0.27	0.19	0.28
*F*	13.43^***^	18.03^***^	29.54^***^	7.16^***^	55.03^***^	10.00^***^	30.84^***^
**Δ*R^2^*		0.54^****^	0.08^***^		0.17^***^		0.10^***^

*CA is short for career adaptability; PP is short for proactive personality. ^∗^p < 0.05; ^∗∗^p < 0.01; ^∗∗∗^p < 0.001; ^+^ indicates that 0.05 < p < 0.1.*

**FIGURE 2:**
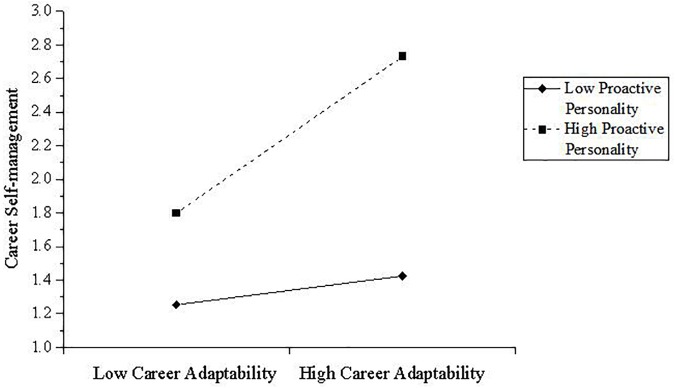
Interaction between career adaptability and proactive personality on career self-management. Low career adaptability and low proactive personality are defined as at least one standard deviation above the mean; high career adaptability and high proactive personality are defined as at least one standard deviation above the mean. High number indicated greater career self-management.

We then tested the moderation hypothesis in our study. Based on Model 1 (i.e., the positive effect of career adaptability on career self-management) in Table 2, we entered proactive personality and the interaction item to the regression model. Results show that (i.e., Model 3 in Table 2) career adaptability (*B* = 0.19, *p* < 0.05), proactive personality (*B* = 0.38, *p* < 0.001) and the interaction item (*B* = 0.24, *p* < 0.001) all have significant positive effects on career self-management. We plotted the interaction at one standard deviation plus and minus the mean of proactive personality, to estimate the nature of the mediator (see Figure 2). Results are as expected, showing a stronger relationship between career adaptability and career self-management when proactive personality is higher, relative to when proactive personality is lower. Therefore, our hypothesis 2 is supported.

Most previous studies have investigated proactive personality as an antecedent of career adaptability in predicting adaptation outcomes, but few studies have examined how proactive personality interacts with career adaptability within the CCT framework (Savickas and Porfeli, 2012). We further made a comparison between our model and what has been studied previously to ascertain which could explain career self-management better. As Table 2 shows, career self-management was taken as the outcome variable in the two models. We first entered control variables (i.e., gender, education, and length of service), career adaptability and proactive personality as independent variables into the regression model 2. Then, we added the interaction item of career adaptability and proactive personality to the regression model 3 (as the competitive model). Results indicate that 26% variance of the outcome could be explained in model 2, whereas 34% variance of the outcome could be explained in model 3. Accordingly, model 3 could explain more variation of career self-management (*ΔR^2^* = 0.08, *p* < 0.01). To conclude, in the CCT framework, when considering career self-management as an adapting behavior and considering proactive personality as an indicator of adaptivity, we need to consider the combined effect between career adaptability and proactive personality, instead of only their separate effects on the adapting behavior.

As a supplementary analysis, we also compared the effects that proactive personality (i.e., adaptivity or will) and career adaptability (i.e., ability) have on – the adaptation result – performance. Therefore, we added two regression models (i.e., model 6 and model 7 in Table 2) with performance as the dependent variable in the two models. The supplementary analysis will help to form a full understanding of how adaptivity (i.e., proactive personality) and adaptability (i.e., career adaptability) in the CCT framework will influence the adaptation result. Results show that when we entered proactive personality, career adaptability and their interaction item into the regression model, proactive personality had a significant positive effect on performance (*B* = 0.32, *p* < 0.001), whereas the effect of career adaptability on performance is marginally significant (*B* = 0.122, *p* = 0.10). The effect of the interaction item is not significant (*B* = 0.05, *p* > 0.05). Further, after career self-management was added in Model 7, the effect of career adaptability became totally insignificant (*B* = 0.05, *p* > 0.10). These results indicate that the relationship between proactive personality and performance is much stronger than that between career adaptability and performance. Moreover, results also indicate that the interaction item (i.e., interaction between career adaptability and proactive personality) can only have an effect on career self-management rather than performance.

After testing the moderation hypothesis, we then tested the med-mod hypothesis in our study. We followed the Preacher’s procedure of testing two regression equations (Preacher et al., 2007). First, a “mediator model” with career self-management as the outcome and a second “dependent variable model” with performance as the outcome (Guan et al., 2015) was used. To simply test the moderation model, there should be a significance in the interactions in the first mediator model as illustrated done above. Then, to test the overall model, the indirect effects from independent variables to the outcome, should vary with different levels of the moderator. With the micro PROCESS (Hayes, 2013, 2017) in SPSS software, we conducted the analysis while controlling for all demographics’ effects. These results are shown in Table 3.

**Table 3:** Moderation and moderated mediation effects for proactive personality on career self-management and performance.

Variable	*B*	*SE*	*t*	*p*
**Dependent variable model with career self-management as dependent variable**				
Constant	2.56	0.18	14.58	<0.001
Gender	0.15	0.06	2.65	<0.05
Education	0.04	0.05	0.94	ns
Length of service	-0.07	0.03	-0.24	ns
Career adaptability	0.19	0.07	2.61	<0.01
Proactive personality	0.37	0.07	5.40	<0.001
Career adaptability × Proactive personality	0.24	0.05	5.11	<0.001
**Dependent variable model with performance as dependent variable**				
Constant	2.26	0.25	9.02	<0.001
Gender	0.01	0.06	0.10	ns
Education	-0.07	0.05	-1.50	ns
Length of service	0.05	0.03	1.82	ns
Career self-management	0.47	0.06	7.42	<0.001
Career adaptability	0.12	0.05	2.20	<0.05
**Conditional indirect effect as a function of proactive personality**				

**Value of proactive personality**	**Career adaptability**			
	**Indirect effect**	**Boot SE**	**Boot LLCI**	**Boot ULCI**

-1 SD (-0.59)	0.02	0.05	-0.07	0.13
+1 SD (0.59)	0.13	0.06	0.04	0.27

*Bootstrap sample size = 5000. Results were reported after controlling for gender, age, education, and length of service.*

**FIGURE 3:**
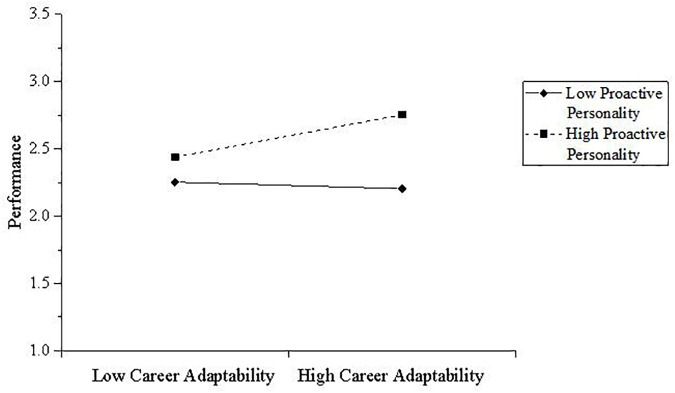
Interaction between career adaptability and proactive personality on performance through career self-management. Low career adaptability and low proactive personality are defined as at least one standard deviation above the mean; high career adaptability and high proactive personality are defined as at least one standard deviation above the mean. High numbers indicate greater career self-management.

The results also support the mod-med hypothesis in our study. Specifically, when the level of proactive personality is higher, there is a significant effect of career adaptability on performance through the mediator, 95% CI = [0.04, 0.27]. In addition, when proactive personality is on a lower level, this indirect effect is not that significant, 95% CI = [-0.07, 0.13] (Figure 3). Thus, Hypothesis 3 is supported.

## Discussion

From the points of the CCT, we investigated the moderating effect that proactive personality has on the relationships between career adaptability, career self-management and performance, among Chinese employees. Specifically, we predicted that career adaptability would positively predict performance (Hypothesis 1a) and career self-management would mediate the positive relationship between career adaptability and performance (Hypothesis 1b). According to the CCT, proactive personality is an operational indicator of adaptivity, career self-management is one of the indicators of adapting responses and performance is viewed as an indicator of adaptation results. Therefore, the two predictions were supported under the CCT framework. These findings are consistent with prior studies addressing the positive influence of career adaptability on performance (Zacher, 2014; Ohme and Zacher, 2015) and the mediating effect of career self-management on the relationship between career adaptability and adaptation results (Rudolph et al., 2017).

We then predicted that, compared with low-level proactive personality group, career adaptability’s effect on career self-management would be stronger for people who have a high level of proactive personality (Hypothesis 2). Further, we supposed that the conditional indirect effect of career adaptability on performance, via self-management, would be stronger for employees who have high as opposed to low levels of proactive personality (Hypothesis 3). Complemented by the conservation of resources theory (Hobfoll, 1989), career adaptability and proactive personality belong to different categories of resources. The role of different resources to a person is the same as the role of different resources to a company. When a company has more resources, such as the support from the government or having many patents, it will be more conducive to gaining benefits. Likewise, the interplay between career adaptability and proactive personality in a person, will produce more proactive results than in the situation when only one kind of resource worked. From the resource’s combination perspective, the two hypotheses were also supported by our findings. These results are also consistent with prior studies indicating that proactive individuals may identify more opportunities (Bateman and Crant, 1993; Crant, 2000) and tend to be more self-initiated to approach career goals than less proactive individuals (Parker et al., 2010). That is, high proactive personality enhances the effects of career adaptability on career self-management behavior, which in turn promotes the improvement of performance. This research identifies proactive personality as a key boundary factor that determines, to what extent, the career adaptability can foster career self-management and performance.

The present study has important theoretical implications. First, these results are in line with the CCT which highlights the mediation role that career self-management plays in the relationship between career adaptability and performance. That is, individuals’ career adaptability will strengthen their performance by enhancing the level of career self-management, which has become one of the main concerns in today’s career field (Baruch et al., 2015). As an indicator of an adapting response, career self-management serves as an important explanatory link in the relationship between adaptability resources and adaptation results under the CCT framework (Savickas, 1997, 2005, 2013). Our study enriches previous studies on career self-management by examining its impact factors as well as its influence on performance in an empirical way.

Second, our use of the resource perspective in conservation of resources theory also deserves reflection. We use the visual of resources in COR to add breadth to our understanding of the relationship between adaptivity and adaptability under the CCT framework. Our findings suggest a new theoretical understanding of the role that proactive personality play. We examine the joint effect of an adaptability resource and adaptivity in predicting adapting responses and adaptation results. In particular, individuals with rich career adaptability and who display more proactive personality, tend to perform well, compared to those who have relatively low levels of career adaptability or are less proactive.

In the present study, we mainly focused on two interaction effects: (1) the interaction effect of proactive personality and career adaptability on career self-management and (2) the interaction effect on the mediation role that career self-management plays between career adaptability and performance. We tested how career adaptability interacts with proactive personality and how their joint effect could affect performance, through the mediation role of career self-management. Results further indicate one’s “will” (i.e., proactive personality, an indicator of adaptivity) strengthens the positive effect of “able” (i.e., career adaptability) on adapting responses as well as the adaptation results. In this way, this study has shed light on the role of adaptivity in the adaptation model of the CCT. From the combination of resources perspective, it is likely that employees who are highly proactive and have a high level of career adaptability, will apply more effort to achieve their career goals, compared to those with only one kind of resource and who do not have high levels of proactive personality. All results indicate that employees’ career adaptability indeed plays a key role in predicting the career self-management which then leads to a good performance. Additionally, proactive personality and career adaptability have a joint effect in predicting career related outcomes. However, the interaction item did not have any effect on the performance outcome. Reasons for this result may be that career adaptability is a career issues-related variable which is affected by each individual, whereas performance is a standard most used by organizations. Research suggests that individuals with proactive personality target proactive work behaviors and achievements (Bateman and Crant, 1993; Crant, 2000). Thus, proactive personality relates more directly with work outcomes than career adaptability and can largely explain the performance.

Results in our study can also inform practice. Both employees and managers need to be aware that proactive personality can strengthen the benefits of career adaptability on enhancing career self-management and improving performance. To increase the effects that proactive personality has on the mediated relationship between career adaptability and performance, some interventions should be implemented to improve employees’ career adaptability and proactivity. For managers, they could set proactive personality as a criterion for selection to ensure newcomers have high levels of proactive personalities, which could be easier to exert the effect of proactive personality on career self-management as well as on the performance. Whereas, for the existing employees, considering that the stable proactive personality is very difficult to change, there may be merit for managers to take measures to improve the employees’ volatile career adaptability resources. For example, increase their career confidence by giving useful and positive feedback or provide them with opportunities to solve some problems; or help them to make clearer career goals to enhance their sense of control and thus improve the overall level of career adaptability.

In addition to the promising results, there are some limitations in the study as well. Participants completed the survey at one time, which may limit the extent to which causal inferences may be made (Podsakoff et al., 2003). Performance is appraised by employees rather than managers, and as with many self-reported survey data, the findings may be affected by the common method variance (Podsakoff et al., 2003). However, Evans (1985) suggests that the common method bias actually has less of an effect on the moderated mediation effect. Future research may use multiple source data or well-designed longitudinal studies to better investigate the causal relationship between all the substantive variables of interest. In addition, the current study took career adaptability as one construct and examined its overall effect in the research model rather than investigating its four dimensions separately. The four dimensions in the career adaptability construct are distinct and future research should pay more attention to specifically investigate the mechanism of each dimension.

In terms of the relationship of all variables we observed, we only found the joint effect of career adaptability and a proactive personality on career self-management but not on performance. The comparison of competitive model in our data analysis further addressed the strong effect of the interplay between career adaptability and proactive personality on career self-management. We regard career adaptability and career self-management as career-related issues, which are more valued by individuals, while performance is more valued by organizations. Given that proactive personality will exert more proactive work behaviors compared to those who are not proactive, the variance of performance will be mostly explained by proactive personality, rather than career related issues like career adaptability and career self-management. Therefore, after proactive personality is added to the model, both career adaptability and the interplay item cannot significantly predict performance. Future studies should pay more attention to the relationship between career related issues and performance.

Despite the enhancement effect that proactive personality plays on career outcomes, proactive personality may potentially also cause some negative results. As a motivational force (Savickas and Porfeli, 2012), proactive personality may exert excessive initiative that could lead to a strong feeling of control. People who are too confident with their abilities and behaviors may have unrealistic expectations of their career. Future studies may examine the negative influence of proactive personality on career outcomes.

Highlighted by the complementing role of conservation of resources theory (Hobfoll, 1989), this study shed light on the interactive effects of adaptivity and adaptability in the CCT framework (Savickas, 1997, 2005, 2013). While this study starts extending the understanding of the relationship between adaptivity and adaptability in the CCT, other aspects of the CCT framework still need to be further investigated. We hope that these results will spur further studies testing the effect of adaptivity on career adaptability in the CCT, to promote individual career development.

## Ethics Statement

An ethics board approval was not required as per institutional guidelines and national laws and regulations since this research did not involve human clinical trials or animal experiments. However, the research was conducted within ethical guidelines. All subjects gave written informed consent in accordance with the Declaration of Helsinki. Respondents were ensured of confidentiality and anonymity, and all participation was voluntary.

## Author Contributions

XG contributed to developing the hypothesis, data analysis, and the manuscripts wright-up. XX contributed to data collection and the first version of manuscript revision. WZ helped form the ideas of this paper and provided suggestions before writing. DJ provided comments on the paper during the revision. All authors listed reviewed and approved this paper for publication.

## Conflict of Interest Statement

The authors declare that the research was conducted in the absence of any commercial or financial relationships that could be construed as a potential conflict of interest.
